# Mitochondria Are Important Determinants of the Aging of Seeds

**DOI:** 10.3390/ijms20071568

**Published:** 2019-03-28

**Authors:** Ewelina Ratajczak, Arleta Małecka, Iwona Ciereszko, Aleksandra M. Staszak

**Affiliations:** 1 Institute of Dendrology, Polish Academy of Sciences, Parkowa 5, 62-035 Kórnik, Poland; 2 Department of Biotechnology, Institute of Molecular Biology and Biotechnology, Adam Mickiewicz University, 61-614 Poznań, Poland; arletam@amu.edu.pl; 3 Plant Physiology Department, Institute of Biology, University of Bialystok, Ciolkowskiego 1J, 15-245 Bialystok, Poland; icier@uwb.edu.pl (I.C.), staszak.a@gmail.com (A.M.S.)

**Keywords:** seeds storage, seed viability, reactive oxygen species, antioxidant system, regulation redox state

## Abstract

Seeds enable plant survival in harsh environmental conditions, and via seeds, genetic information is transferred from parents to the new generation; this stage provides an opportunity for sessile plants to settle in new territories. However, seed viability decreases over long-term storage due to seed aging. For the effective conservation of gene resources, e.g., in gene banks, it is necessary to understand the causes of decreases in seed viability, not only where the aging process is initiated in seeds but also the sequence of events of this process. Mitochondria are the main source of reactive oxygen species (ROS) production, so they are more quickly and strongly exposed to oxidative damage than other organelles. The mitochondrial antioxidant system is also less active than the antioxidant systems of other organelles, thus such mitochondrial ‘defects’ can strongly affect various cell processes, including seed aging, which we discuss in this paper.

## 1. Aging Seeds

Seed aging, which decreases seed viability during storage, is a major problem for successful plant growth and productivity and leads to seed deterioration. In agricultural production, aged seeds cause commercial and genetic losses. The seed aging process is dependent primarily upon the moisture content of seeds, the oxygen level and the temperature at which seeds are stored [1,2,3,4] but is also associated with various metabolic and biophysical seed conditions [3,5]. Although the mechanisms of seed aging are still under intensive study, reactive oxygen species (ROS) are considered the main factor contributing to seed aging [6]. According to the free radical theory of aging, the generation and quantity of ROS are crucial for the progression of seed aging and other aging-associated disorders [7]. During the storage of seeds, the accumulation of ROS leads to the damage of lipids, DNA and proteins and consequently contributes to decreased germination and the loss of seed vigor [6,8,9,10,11,12,13]. It is claimed that for the effective conservation of genetic resources, it is necessary to identify the factors influencing aging of seeds that differ in sensitivity to drying and long-term storage. Based on their properties, seeds are divided into three categories: Orthodox (tolerating drying to a moisture content <7% and storage at −10 °C), recalcitrant (sensitive to drying to a moisture content <27% and conventional storage conditions), and intermediate (loosing viability relatively quickly, compared to orthodox seeds) [14,15]. These seed categories can also display various aging process timings or mechanisms.

## 2. Mitochondrial Activity in Aging Seeds

Mitochondria are the most important source of energy for cell growth and metabolism. In addition, mitochondria are the major sites for the production of reactive oxygen species (mtROS), and thus, are involved in the maintenance of ROS homeostasis [16,17,18,19]. ROS such as superoxide, hydrogen peroxide, and hydroxyl radicals, which oxidize cellular constituents, compromise organellar membrane integrity [20] and the main cell components [21,22]. The superoxide anion (O_2_^−^) is the precursor of most ROS and a mediator in oxidative chain reactions. Furthermore, O_2_^−^ can dismute to produce hydrogen peroxide (H_2_O_2_) and may react with other radicals, including NO. The product of this reaction, peroxynitrite (ONOO^−^), is also a very powerful oxidant [23]. Hydrogen peroxide can freely migrate across cell membranes and can generate highly aggressive HO˙ [6,24]. mtROS are generated, in particular, in the context of respiratory electron transport activity. The main mtROS production sites are complex I and complex II of the mitochondrial electron transport chain (ETC). ETC-mediated ROS generation is primarily due to the presence of ubisemiquinone radicals, which can transfer a single electron to oxygen, giving rise to the production of superoxides [25]. In plants, alternative oxidase (AOX) plays a key role during stress by lowering ROS production from the ETC by preventing excessive reduction of the mitochondrial ubiquinone pool [20].

However, the relationship between the AOX pathway and ROS accumulation in the mitochondria of aged seeds has not yet been thoroughly studied [26]. In mitochondria, there are proteins that cause ROS generation: Glycerolphosphate dehydrogenase [27], multi-subunit pyruvate dehydrogenase complex and a structurally similar membrane-bound enzyme complex of alfa-ketoglutarate dehydrogenase (alfa-KGDH) [28]. Researchers have reported that succinate dehydrogenase (SDH) also contributes to mtROS production [26,29]. Aconitase, an enzyme in the mitochondrial matrix, is able to transform hydrogen peroxide into hydroxyl radicals during a Fenton reaction in the presence of iron and sulfur [30]. ROS are generated not only in the mitochondria but also by NADPH oxidase in the conversion of NADPH to NADP^+^ [23]. ROS are produced during germination in multiple species, where they have been proposed as a signal for release from seed dormancy [20].

Additionally, mitochondria are a source of reactive nitrogen species derived from nitric oxide (NO^·^). NO^·^ is generated enzymatically by a family of nitric oxide synthases (NOS). These enzymes synthesize NO^·^ using L-arginine as a substrate and NADPH as an electron source in the presence of Ca^2+^ ions and reduced thiols [21]. Mitochondrial electron transport can also produce NO [31,32]. NO has a short half-life but can react with thiols or/and the catalytic metal center of proteins, which results in the covalent modification of cysteine residues, termed S-nitrosylation. This modification also regulates many cellular processes in seeds/germination [31,32,33,34].

## 3. Oxidative Damage in Aging Seed Mitochondria

ROS-related mitochondrial dysfunction plays a vital role in seed deterioration, but the detailed mechanism of this role remains largely unknown. Most studies are based on the seeds of herbaceous plants (Table 1), and seeds of one tree species. The past ten years brings much more knowledge in this topic than could have been expected.

The accumulation of ROS causes oxidative damage and dysfunction and membrane system disorders, as well as the oxidative damage of mitochondrial proteins, DNA and lipids [45]. Oxidative damage of mitochondrial proteins include damage to subunits of the pyruvate decarboxylase complex, subunits of ATP synthase, and enzymes of the tricarboxylic acid (TCA) cycle [46]. ROS cause direct oxidation of amino acids, oxidation of Cys residues, which form disulphide bonds, oxidation of Met residues, which forms Met sulphoxide, and oxidation of arginine, lysine, proline, histidine, serine and threonine residues, which creates carbonyl groups in side chains [23,47]. The activity of mitochondrial proteins is regulated at the post-translational level, among others, e.g., S-nitrosylation [48,49]. During oxidative stress, the activity of mitochondrial proteins is lowered by the binding of lipid peroxidation products [50], carbonyl group formation [51] and the oxidation of tryptophan residues [52]. In elm seeds (*Ulmus* L.) 48 mitochondrial proteins changed during aging and found that these changes were associated with the tricarboxylic acid cycle (TCA) and mitochondrial ETC [44]. During oat seed aging, Moa et al. [26] also showed that proteins in the TCA cycle were down-regulated, and several enzymes related to mitochondrial ETC were up-regulated. Other authors [53] showed that the most recognized source of mtDNA mutagenesis are ROS, that are produced by the ETC. In addition, H_2_O_2_ induced strand breaks and abasic sites in mtDNA. The damage of main cell components by ROS leads to mitochondrial dysfunction through Bax induction and cytochrome c release [23].

Lipid peroxidation in the mitochondrial membranes refers to the free radical peroxidation of the polyunsaturated fatty acids of membrane lipids [54]. Malondialdehyde (MDA) and 4-hydroxy-2-nonenal (HNE) are products of lipid peroxidation and can interact with cells to reduce or even eliminate their functions. MDA can react with DNA bases, resulting in gene mutations, and HNE reacts mostly with proteins, leading to functional alterations [23]. Increases in mitochondrial MDA and H_2_O_2_ contents in moist tissues damage the mitochondrial membrane structure and their function [55].

The accumulation of oxidative damage is the basis of Harman’s free radical theory of aging [56]. One of the main sources of ROS in the cell is oxidative phosphorylation within mitochondria, so the free radical theory of aging may essentially be a mitochondrial theory of aging for plant seeds.

## 4. The Antioxidative System in Aging Seed Mitochondria

The balance between ROS generation and detoxification by antioxidants modulates the redox reactions in plant cells. Respiration and various other metabolic processes, including responses to oxidative stress, are co-controlled by the cellular redox state [57,58]. The plant mitochondrial matrix contains ROS scavenging systems, including enzymatic and nonenzymatic antioxidative systems [40], such as the manganese-superoxide dismutase (Mn-SOD) and ascorbate-glutathione (ASA-GSH) cycles [42], catalase (CAT) and peroxiredoxin (including peroxiredoxin IIF) [59]. During the aging of seeds, a decrease in the mitochondrial ASA-GSH cycle results in less reduced/oxidized forms of ASA and GSH, which might lead to ROS accumulation, affecting mitochondrial dysfunction [40]. Xia et al. showed in aging soybean seed mitochondria, that SOD, ascorbate peroxidases (APX), monodehydroascorbate reductase (MDHAR), and glutathione reductase (GR) activities decreased with prolonged aging.

Mao et al. [26] suggested that mitochondrial structure changes are responsible for decreases in antioxidant enzyme activity in aged seeds. The authors observed decreases in the activities of GR, dehydroascorbate reductase (DHAR) and MDHAR, which were accompanied by damage to the inner mitochondrial membrane in seeds during aging [26,42]. The observed catalase (CAT) is believed to be associated with the mechanism of seed aging [6,60], which actively removes H_2_O_2_ [61] and modulates the associated signaling pathways [62,63]. CAT levels decrease in the mitochondria of *Fagus sylvatica* L. seeds during their natural aging process. The decrease in the activity of this enzyme is accompanied by an increase in the H_2_O_2_ level (Ratajczak et al., results not published).

The active adjustment of the redox state in the mitochondria is important for the normal course of the combined metabolic processes of photosynthesis and respiration in green tissues. Ascorbate (ASA) and glutathione (GSH) play an important role in regulating the redox state in the mitochondria. Moreover, they act together in the ASA–GSH cycle, which is a core component of the antioxidant system in plant mitochondria, and have other functions that are important for the mitochondrion. ASA also participates in redox regulation in the modulation of gene expression, in the regulation of enzymatic activity, and in cell signaling [4,64,65,66,67,68]. Changes in mitochondrial ascorbate synthesis may modulate communication between plastids and mitochondria [65,69]. Mitochondrial GSH levels depend on the transport of glutathione into the mitochondria because GSH is mainly synthesized in the cytosol and plastids [70]. There is little information about plant mitochondrial glutathione transporters. The level of mitochondrial GSH is highly dependent on the activity of the enzyme glutathione reductase (GR), which reduces GSH to its oxidized form glutathione (GSSG) [71]. GSH deficiency in the mitochondria can cause mitochondrial damage, affecting changes in the synthesis of thiol proteins and thus changes in redox regulation in mitochondrion cells. The potential E = 2GSH/GSSG redox state in mitochondria, which ranges from –340 to −300 mV, provides a unique environment that affects the corresponding thiol modification of proteins in the mitochondria [69].

ROS that are produced in the mitochondrial matrix are detoxified by thiol-based peroxidase systems, such as peroxiredoxin (Prx) [72]. Prx family members have an important role in regulating and maintaining the redox balance in seed cells [73,74]. Plant mitochondria contain an atypical type II Prx (Prx IIF) [25,59,75]. Prx IIF is a peroxidase that accepts electrons from a broad range of donors and functions principally in the reduction H_2_O_2_, which catalyzes the detoxification of ROS in the following order of efficiency: H_2_O_2v_ > tertiary butyl hydroperoxide (-BOOH) > cumene hydroperoxide (CuCOOH) [75,76].

We found that the level of the protein Prx IIF decreased in the mitochondria of beech (*Fagus sylvatica* L.) seeds during the natural aging process (Ratajczak, results not published). We also observed differences in Prx IIF transcript and protein levels, as well as in the level of post-translational modification between Norway maple (*Acer platanoides* L.) seeds (orthodox seeds) and sycamore (*A. pseudoplatanus* L.) seeds (recalcitrant seeds) during desiccation [77]. It has been proposed that the redox homeostasis of mitochondria in seeds is a necessary feature to maintain high seed viability.

**Figure 1:**
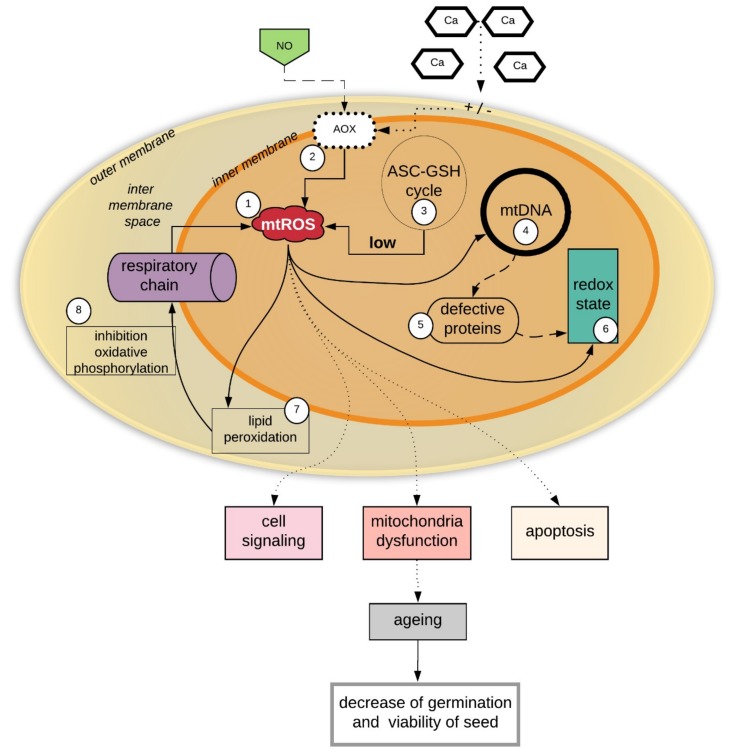
Dysfunctional mitochondria and aging of seeds. Due to continuous reactive oxygen species (ROS) production in mitochondria, the level of ROS increases during seed storage (1). The nitrogen oxide (NO) influence on alternative oxidase (AOX) (2), which cause ROS generation (2). Calcium ions change membrane potentials and are also influenced by AOX (2) on the increase of ROS level in mitochondria. The ascorbate-glutathione cycle (3) does not effectively remove ROS (3), which causes oxidative damage to mtDNA (4) and protein synthesis (5) and leads to changes in signaling and the redox status in the mitochondria (6). Increasing ROS levels (7) in the mitochondria cause oxidative damage to the membranes (7), which affects the inhibition of oxidative phosphorylation (8). All of these events cause a decrease in seed viability and show the basis of aspects of seed aging. Solid arrows shows the process that happened in ageing seeds, the doted arrows explain in which processes ROS participate, detailing processes of ageing and decrease of germination and viability of seed.

Another possible regulator of the redox state in plant mitochondria is the thioredoxin (Trx) system [78]. The Trx system consists of o-typ Trx (Trx-o) and NADPH-thioredoxin reductase (Figure 1) [79,80]. Thioredoxins (Trx), a regulatory disulfide protein, is a substrate for enzymes that catalyze reactions [78] and regulatory reactions that alter the activity or other functional properties of interaction target proteins [81]. The reduced form of Trx interacts with a variety of target proteins and performs regulatory functions [82].

Members of the Trx system in higher plants are divided into groups: m, f, x, y, o and h [83,84]. Trx o is localized in the mitochondria, and Trx h is typically cytosolic but has also been identified in other cellular compartments, including the mitochondrion [84]. Trx participates in the regulation of 12 mitochondrial processes, ranging from energetics and metabolism reactions to protein synthesis, stress responses and communication with other organelles [79,85]. It has been proposed that Trx functions not only to regulate biochemical processes under optimal conditions but also to restore the function of activities after oxidative stress (adaptation to stress). Sanz-Barrio et al. [86] showed that Trx acts in plant mitochondria as a molecular chaperone. The role of Trx in the regulation of the redox state in tree seeds, which is characterized by a different sensitivity to water loss, is not yet fully understood.

## 5. Is Mitochondrial Dysfunction the Cause of Seed Aging?

The functions of mitochondria in cell signaling events and inter-organelle communication and aging are already well known, especially in animal cells [87,88]. The role of mitochondria in the plant seed aging processes has not yet been well described. It is important to determine whether mitochondrial dysfunction imitates seed aging and whether mitochondrial dysfunction is the result of seed aging during storage. It is believed that the dysfunction of mitochondria, coupled with plant seed aging, is as is presented in Figure 1.

**Figure 2:**
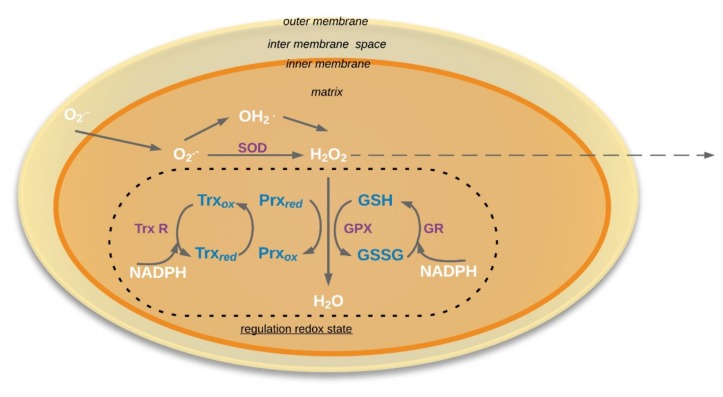
Schema of regulation redox state in the mitochondria. O_2_^•−^: Superoxide; HO_2_˙: Hydroperoxyl radical; SOD: Superoxide dismutase (E.C. 1.15.1.1); GR: Glutathione reductase (EC 1.6.4.2); GSH: reduced form glutathione; GSSG: oxidized form glutathione; GPX: glutathione peroxidases (EC 1.11.1.9); Prx: peroxiredoxin; Trx: Thioredoxin; TrxR: NADPH-thioredoxin reductase (EC 1.8.1.9). Solid arrows mean that process occurred in mitochondria, dotted arrows mean that molecules could be exported outside the mitochondria.

The effects of mitochondrial damage in seeds during storage, which finally leads to seed aging and the reduction of seed viability, are summarized in Figure 1. (1) Mitochondria are the sites of continuous ROS generation. (3) During the storage of seeds, the ROS level increases, mainly in the mitochondria, and the excess of ROS may not be effectively removed due to the low activity of the mitochondrial antioxidant system (i.e., the ASA-GSH cycle) compared to the antioxidant systems of other cell organelles. The ROS-induced impairment of mitochondria leads to increased oxidant production and oxidative damage. A high level of ROS in the mitochondria causes oxidative damage to the membranes, which affects the inhibition of oxidative phosphorylation. (4) ROS also cause oxidative damage to mtDNA. mtDNA is constantly exposed to oxidative injury, mainly due to the location of mtDNA in the inner mitochondrial membrane, which exposes it to the influence of ROS and makes mtDNA more exposed than nuclear DNA to oxidative damage [89]. Most studies on aging concern human cells, while research on the mtDNA in plant seeds is uncommon. However, damage to plant seed mtDNA is a very important problem for genome reserve conservation because a lack of genome integrity affects seed viability [90]. The stability of mtDNA depends on the production of mitochondrial ROS (mtROS), which are generated during normal electron flux via mitochondrial electron transport [89,91]. mtDNA is not protected by the protein membrane, and histones are not associated with mtDNA it, which makes mtDNA more sensitive to increases in ROS levels [92]. In aging seeds, as was shown in Figure 2, the accumulated mtROS (1) induces damage to the mtDNA (4) and adversely affects the synthesis of mitochondrial proteins (5), including proteins regulating the mitochondrion redox state, e.g., peroxyredoxins (Prxs) and thioredoxins (Trxs). The accumulation of mtROS thus influences changes in signaling and the redox status in the mitochondria (6). (7) A high level of ROS in the mitochondria causes oxidative damage to membranes, which affects the inhibition of oxidative phosphorylation (8). The sum of all adverse reactions (1–8) causes a decrease in seed viability (Figure 1).

The cellular ROS levels and redox status of mitochondria regulate mitochondrial and nuclear gene expression, which are pivotal to seed aging [7,76]. Generally, changes in the mitochondrial redox state will affect not only mitochondrial activity but also cellular processes, such as photosynthesis, stress defenses and the activation of programmed cell death [76].

One potential regulator of mitochondrial activity during aging is the second messenger Ca^2+^. By stimulating Ca^2+^-dependent dehydrogenases of the tricarboxylic acid (TCA) cycle, Ca^2+^ boosts the activity of the mitochondrial respiration chain and, consequently, the mitochondrial adenosine triphosphate (ATP) production via oxidative phosphorylation. By stimulating the mitochondrial respiration chain, matrix Ca^2+^ also maintains the stability of the mitochondrial membrane potential, which is temporarily dissipated by entering Ca^2+^. However, in the case of overwhelming mitochondrial Ca^2+^ accumulation, the permeability of the inner mitochondrial membrane increases drastically, resulting in the dissipation of the mitochondrial membrane potential, the shutdown of mitochondrial respiration and finally, the initiation of cell death signaling pathways [93]. Wang and coauthors [94] indicated that the Ca^2+^ flux could be part of the AOX retrograde response.

Xin et al. showed that aging severely affects the rate of NADH and succinate-dependent O_2_ consumption and the respiration control rate, suggesting that aged seeds possess a lower capacity than control seeds for the electron transport chain. Aging directly reduces the efficiency of electron transport chains, thereby reducing ATP production, so aged seeds cannot provide sufficient ATP for germination. Upon stress exposure in mitochondria, an energy deficit signal occurs, which leads to global changes in organellar and nuclear gene expression [20].

## 6. Mitochondrial Structure in the Process of Seed Aging

The presence of two membranes—the outer membrane (OM) and inner membrane (IM)—with very different compositions and conformations, suggests that the membranes have diverse contributions to mitochondrial function and physiology. Mitochondria modulate their functions and status and allow complex quality control. Recent discoveries have shown a correlation between the modulation of mitochondrial shape and network and the energetic state of the cell. Oxidative stress causes mitochondrial elongation, protecting mitochondria from degradation and promoting mitochondrial ATP production [95]. Xia et al. suggested there are relationships between antioxidative systems and and mitochondrial ultrastructure in aging seeds. The authors of this study used transmission electron microscopy to observe that mitochondrial ultrastructure was damaged in aging and the degree of damage was related to the level of seed moisture. Increasing the moisture content from 4% to 16% in the seeds at 0, 16 and 40 days caused the cristae to no longer be visible. Others authors [44], by using fluorescence microscopy, showed that the mitochondrial distribution and morphology changes gradually with seed aging in *Ulmus pumila* L. These authors noticed that mitochondrial aggregation in the early aging stage is related to mitochondrial endogenous ROS production. Yin and co-authors indicated that the integrity was highly inhibited in rice embryos aged seven days, in comparison to rice embryos aged 0 and 4 days, and the oldest embryos possessed numerous mature mitochondria with typical structures of well-developed cristae.

Using electron microscopy, Noctor et al. [96] showed that mitochondria, which have elaborate cristae in young and mature Arabidopsis rosette tissues, only lose their internal structure with swollen cristae at the final programed cell death (PCD) stage of senescence, when most cellular proteins and other reserves have been degraded and exported. Electron cryotomography of mitochondria isolated from aging *Podospora anserina* revealed a sequence of events, namely, the progressive vesiculation of the mitochondrial inner membrane, the collapse of the cristae, disassembly of ATP synthase dimers, and formation of large contact sites between the inner and outer mitochondrial membranes [97].

It is likely that changes in the structure of mitochondria caused by ROS generation and mitochondrial dysfunctions trigger different responses that regulate mitochondrial and nuclear gene expression, which are pivotal to seed aging.

## 7. Conclusions

We believe that a thorough analysis of seed mitochondria in various tissues will bring us closer to understanding the causes of the seed aging process. It is important to perform these analyses on seeds that have different sensitivities to water loss, storage conditions and long-term storage, i.e., in orthodox, recalcitrant and intermediate seeds. We suggest that more damage to mitochondria will occur in the embryonic axes than in the cotyledons of seeds.

The mitochondria are very important cellular organelles, whose main purpose is to generate energy in the process of cellular respiration—in the form of ATP—during the process of cellular respiration. In mitochondria, seed aging increases the level of ROS, which causes numerous organelle dysfunctions. This contributes to increases in the level of oxidative damage to the main cellular components, decreases in enzyme activities due to the oxidation of the functional groups, and increases membrane lipid peroxidation. During the aging of seeds, ROS initiates gene expression, which is responsible for programmed cell death. In addition, physiological and biochemical changes affect changes in the structures of mitochondria, which determines the action of these organelles. Mitochondria are well-suited for sensing functional imbalances. Their respiratory machinery, which is based on redox chemistry, can react sensitively to changing conditions. At the final stage of mitochondrial aging, the disruption of this arrangement results in the ability of mitochondria to produce ATP. In effect, aging cells with an increasing proportion of dysfunctional mitochondria are less fit than non-aging cells and eventually die. Understanding the mechanisms of seed aging will lead to new methods for seed conservation and longevity.

## Figures and Tables

**Table 1:** The role of reactive oxygen species in dysfunction mitochondrion seeds.

Organism (Species)	Processes	References
Pea (*Pisum sativum* L.)	Germination after *Aspergillus ruber* infection, aging	[35]
Rye (*Secale cereal* L.)	Embryogenesis, germination	[36]
Soybean [*Glycine max* (L.) Merr]	Aging	[37]
Pea (*Pisum sativum*)	Germination	[25]
Soybean [*Glycine max* (L.) Merr]	Germination, imbibition	[38]
Pea (*Pisum sativum* cv. Jizhuang)	Germination	[39]
Soybean [*G.max* (L.) Merr]	Aging	[40]
Elm (*Ulmus pumila* L.)	Aging	[41]
Oat (*Avena sativa* L.)	Aging	[42]
Rice (*Oryza sativa* L.)	Aging	[7]
Rice (*Oryza sativa* L.)	Aging	[43]
Elm (*Ulmus pumila* L.)	Aging	[44]
Oat (*Avena sativa* L.)	Aging	[26]
